# Analyzing the influence of kinase inhibitors on DNA repair by differential proteomics of chromatin-interacting proteins and nuclear phospho-proteins

**DOI:** 10.18632/oncotarget.22424

**Published:** 2017-11-10

**Authors:** Lisa Gleißner, Marcel Kwiatkowski, Laura Myllynen, Pascal Steffen, Cordula Petersen, Kai Rothkamm, Hartmut Schlüter, Malte Kriegs

**Affiliations:** ^1^ Laboratory of Radiobiology and Experimental Radiation Oncology, Hubertus Wald Tumorzentrum, University Cancer Center Hamburg, University Medical Center Hamburg, Eppendorf, Hamburg, Germany; ^2^ Institute of Clinical Chemistry, Hubertus Wald Tumorzentrum, University Cancer Center Hamburg, University Medical Center Hamburg, Eppendorf, Hamburg, Germany; ^3^ Department of Pharmacokinetics, Toxicology and Targeting, Groningen Research Institute for Pharmacy, University of Groningen, Groningen, The Netherlands

**Keywords:** differential proteomics, phospho-proteins, chromatin bound proteins, sorafenib, head and neck cancer

## Abstract

The combination of radiotherapy and pharmacological inhibition of cellular signal transduction pathways offers promising strategies for enhanced cancer cell inactivation. However, the molecular effects of kinase inhibitors especially on DNA damage detection and repair after X-irradiation have to be understood to facilitate the development of efficient and personalized treatment regimens. Therefore, we applied differential proteomics for analyzing inhibitor-induced changes in either chromatin-bound or phosphorylated nuclear proteins. The effect of the multi kinase inhibitor sorafenib on DNA repair, chromatin binding and phosphorylation of nuclear proteins was analyzed in UT-SCC 42B head and neck cancer cells using metabolic labeling based differential proteomics (SILAC). Sorafenib significantly inhibited DNA repair but failed to significantly affect chromatin interactions of 90 quantified proteins. In contrast, analyzing nuclear phospho-proteins following sorafenib treatment, we detected quantitative changes in 9 out of 59 proteins, including DNA-repair proteins. In conclusion, the analysis of nuclear phospho-proteins by differential proteomics is an effective tool for determining the molecular effects of kinase inhibitors on X-irradiated cells. Analyzing chromatin binding might be less promising.

## INTRODUCTION

To improve cancer treatment radiotherapy is combined with radiation response modifiers (radiosensitizers). In this context numerous kinase inhibitors blocking important cellular signal transduction pathways such as EGFR-, mTOR or Raf-signaling have been tested over the past years in combination with X-irradiation (IR) [1]. The most prominent example is the combination of radiotherapy with cetuximab, an inhibitory antibody directed against the epidermal growth factor receptor (EGFR) which is used to treat patients with head and neck squamous cell carcinoma (HNSCC; [2]). Many other antibodies and small molecule inhibitors have been tested in combination with IR in pre-clinical as well as clinical studies with partly promising results [1]. However, the main obstacle of this targeted treatment is the heterogeneity in respect to response, which makes response prediction essential. However, for effective prediction the molecular effects of the inhibitors on the irradiated cells have to be understood.

The most critical lesions induced by X-irradiation are DNA lesions, especially DNA double stand breaks (DSB). If DNA DSB are not repaired properly, lethal chromosomal aberrations might occur [3]. Therefore, cancer cell-specific inhibition of DNA repair is one of the most wanted effects, if targeted agents are combined with IR and many kinase inhibitors have already been shown to efficiently inhibit DNA DSB repair [4–9]. But still, the exact molecular mechanisms are usually unknown which is due to the high complexity of DNA damage detection and repair.

Irradiation induces a DNA damage response (DDR). This DDR includes the activation of kinases such ATM, DNA-PKcs or ATR, leading to extensive phosphorylation of hundreds of proteins involved in cell cycle regulation, apoptosis, chromatin remodeling and especially DNA repair [10, 11]. Besides protein phosphorylation, additional posttranslational modifications such as ubiquitylation, SUMOylation, acetylation or PARylation and the recruitment of DNA proteins to the site of damage are of central importance [10]. Since kinase inhibitors — which are not primarily directed against kinases involved in the DDR but against those essential for signal transduction such as EGFR, AKT or Raf — might influence any of these mechanisms, differential proteomic approaches have to be established to analyze their influence on these different mechanisms in DDR and DNA repair.

So far most studies which used differential spatial proteomics focused on protein phosphorylation, acetylation or ubiquitylation after DNA damage induction only, with a few studies also analyzing the recruitment of repair proteins to the damaged DNA [10] or the re-localization of nuclear proteins [12, 13]. Even fewer studies have analyzed the influence of kinase inhibitor treatment on IR-induced DDR and DNA repair. To address this question we have recently established a quantitative mass spectrometry (MS) approach to analyze changes in nuclear protein phosphorylation. Using this approach we have already successfully unveiled the mechanism by which EGFR inhibition affects DNA DSB repair in HNSCC cells, demonstrating the effectiveness of this approach [14]. In the present study, we compare this phospho-proteomic approach with an approach analyzing proteins, which are altered in their interaction with the chromatin. We used the HNSCC cell line UT-SCC 42B cells which were treated with the multi kinase inhibitor sorafenib since we have already reported radiosensitization and inhibition of DNA DSB in HNSCC cells by sorafenib [7, 15]. Following sorafenib treatment we detected several changes regarding the nuclear phospho-proteins. However, no changes were observed in the chromatin fraction, which indicates that analysis of the phospho-proteome might be more promising to unveil the molecular mechanism of action of kinase inhibitors in respect to DDR and DNA repair.

## RESULTS

The aim of this study was to compare two quantitative MS approaches analyzing either the chromatin binding or the phosphorylation of nuclear proteins after kinase inhibitor treatment. For this purpose, we used the HNSCC cell lines UT-SCC 42B and the multi kinase inhibitor sorafenib, which has already been shown to inhibit DNA DSB repair and to increase the radiosensitivity of UT-SCC 42B cells [7].

### Inhibition of DNA double strand break repair by sorafenib

**Figure 1:**
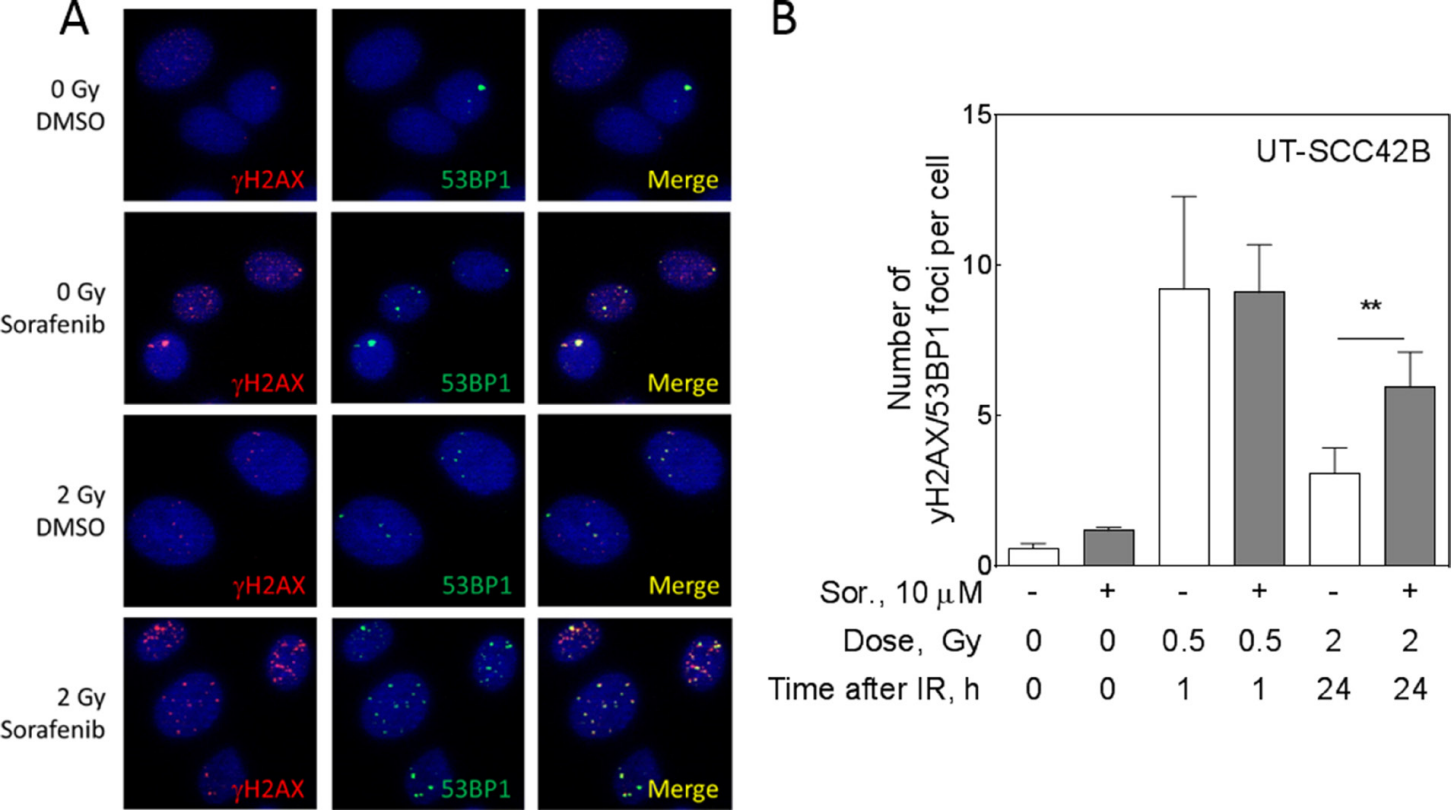
Sorafenib inhibits DSB repair in UT-SCC 42B cells Cells were treated with sorafenib 2 h before IR and DSB repair was analyzed using immunofluorescence staining of γH2AX/53BP1 foci either 1 h after 0.5 Gy (DSB induction) or 24 h after 2 Gy of IR (repair). (**A**) Sample pictures of residual foci 24 h after 2 Gy (red, gH2AX; green, 53BP1; merge, yellow; DNA/DAPI, blue). (**B**) Quantification.

To prove DNA repair inhibition by sorafenib in our experimental setting, we analyzed residual DNA DSB by detecting γH2AX and 53BP1 double-positive repair foci using immunfluorescence microsopy (Figure 1). UT-SCC 42B cells were treated with 10 µM sorafenib for 2 h before irradiation with 2 Gy. The next day the cells were replated and stained for γH2AX/53BP1 foci 24 h later (Figure 1A). The quantification of four independent experiments revealed significantly impaired DNA DSB repair, since γH2AX/53BP1 foci were increased by sorafenib (Figure 1B; *p* = 0.044), which is in perfect agreement with our previously published data [7]. This increase was not caused by an elevated number of induced DSB, as demonstrated by similar numbers of DSB after 1 h induced by 0.5 Gy (Figure 1A).

### Labeling of cells

**Figure 2:**
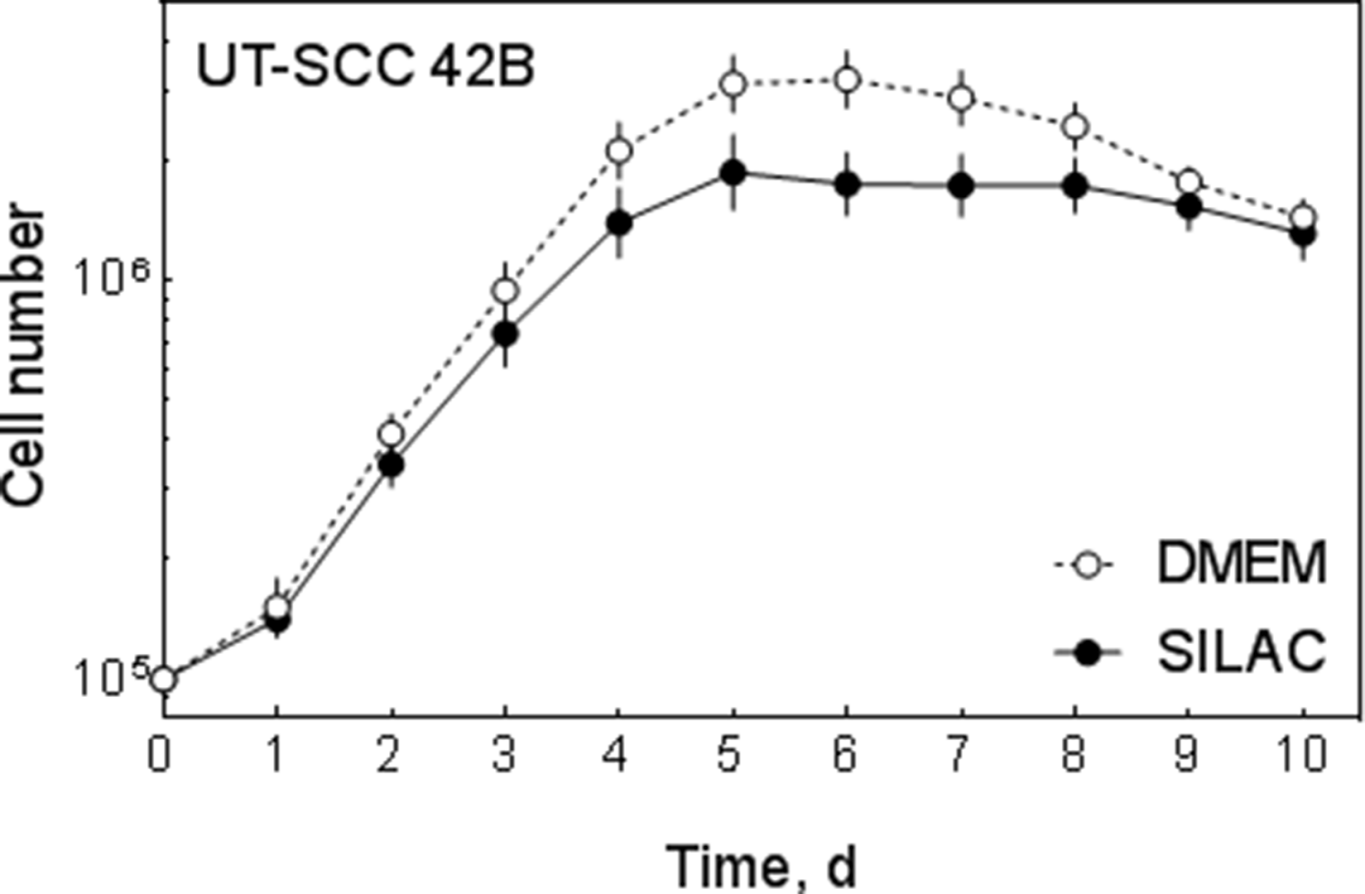
Cell growth under SILAC conditions Growth of UT-SCC 42B cells was determined by analyzing the cell number for 10 days under normal (DMEM) and SILAC-conditions.

To enable a quantitative analysis of changes induced by sorafenib we used stable isotope labeling with amino acids (SILAC). To this end we cultured UT-SCC 42B cells in the presence of either light (L) [^12^C_6_] L-arginine/[^12^C_6_] L-lysine or heavy (H) [^13^C_6_] L-arginine/[^13^C_6_] L-lysine. Since SILAC medium contains dialyzed foetal bovine serum (FBS) which might reduce proliferation, we analyzed the proliferation of the cells by determining the cell number up to 10 days. As shown in Figure 2 UT-SCC 42B cells were able to grow under SILAC conditions with a slightly reduced proliferation rate.

**Figure 3:**
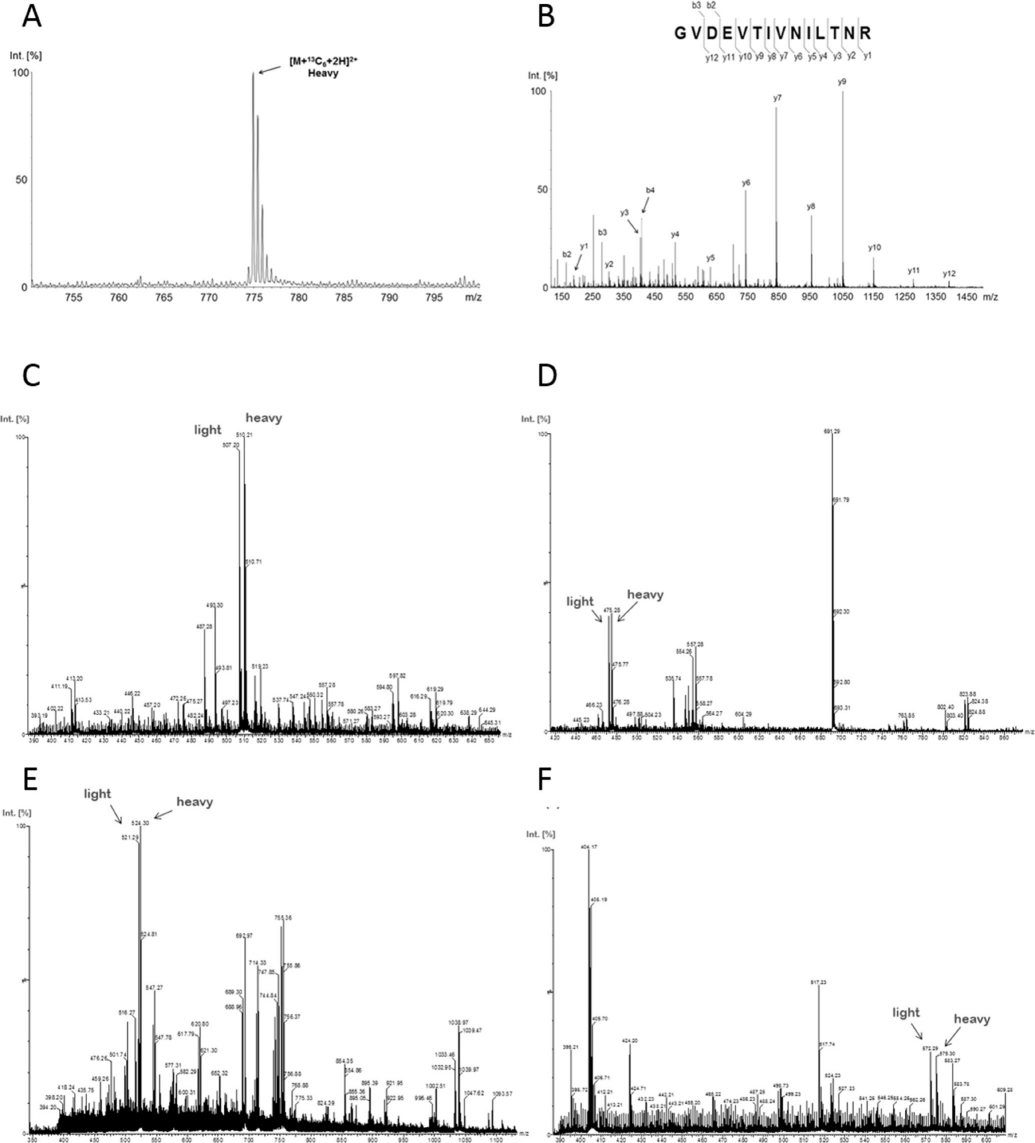
Incorporation of amino acids labeled with stable isotopes (13C6) After five doublings in SILAC media, the incorporation of the stable isotopes was analyzed by MS. (**A**) The complete incorporation is exemplarily demonstrated by the precursor MS spectrum of the GVDEVTIVNILTNR(13C6) peptide from the protein Annexin A2. The intact peptide precursor-spectrum shows only a signal for the completely stable isotope labelled peptide ion (heavy) indicating a full incorporation of the stable isotopes. (**B**) Fragment-spectrum with annotated b- and y-ion series of the stable labeled peptide GVDEVTIVNILTNR(13C6). (**C**–**F**) The precursor MS spectra exemplarily represent high abundant peptides (C: GGNFGFGDSR, ROA2_HUMAN; E: VLQSALAAIR, ILF2_HUMAN) and a low abundant peptides (D: VPPPPPIAR, HNRPC_HUMAN; F: FATHAAALSVR; SFPQ_HUMAN) with an almost 1:1 ratio for none-labeled (light) and stable isotope labeled (heavy) peptides. This further highlights the complete incorporation of stable isotopes and that equal protein amounts of none-labeled and stable isotope labeled cells were combined for differential LC-MS analysis.

Furthermore, incorporation of the labled aminoacids was complete after five passages as proven by MS analysis of nucelar extracts (Figure 3). Therefore for all further experiments cells were used which had been cultivated at least five passages in SILAC medium.

### Quantitative analysis of chromatin binding and phosphorylation of nuclear proteins by SILAC-MS

**Figure 4:**
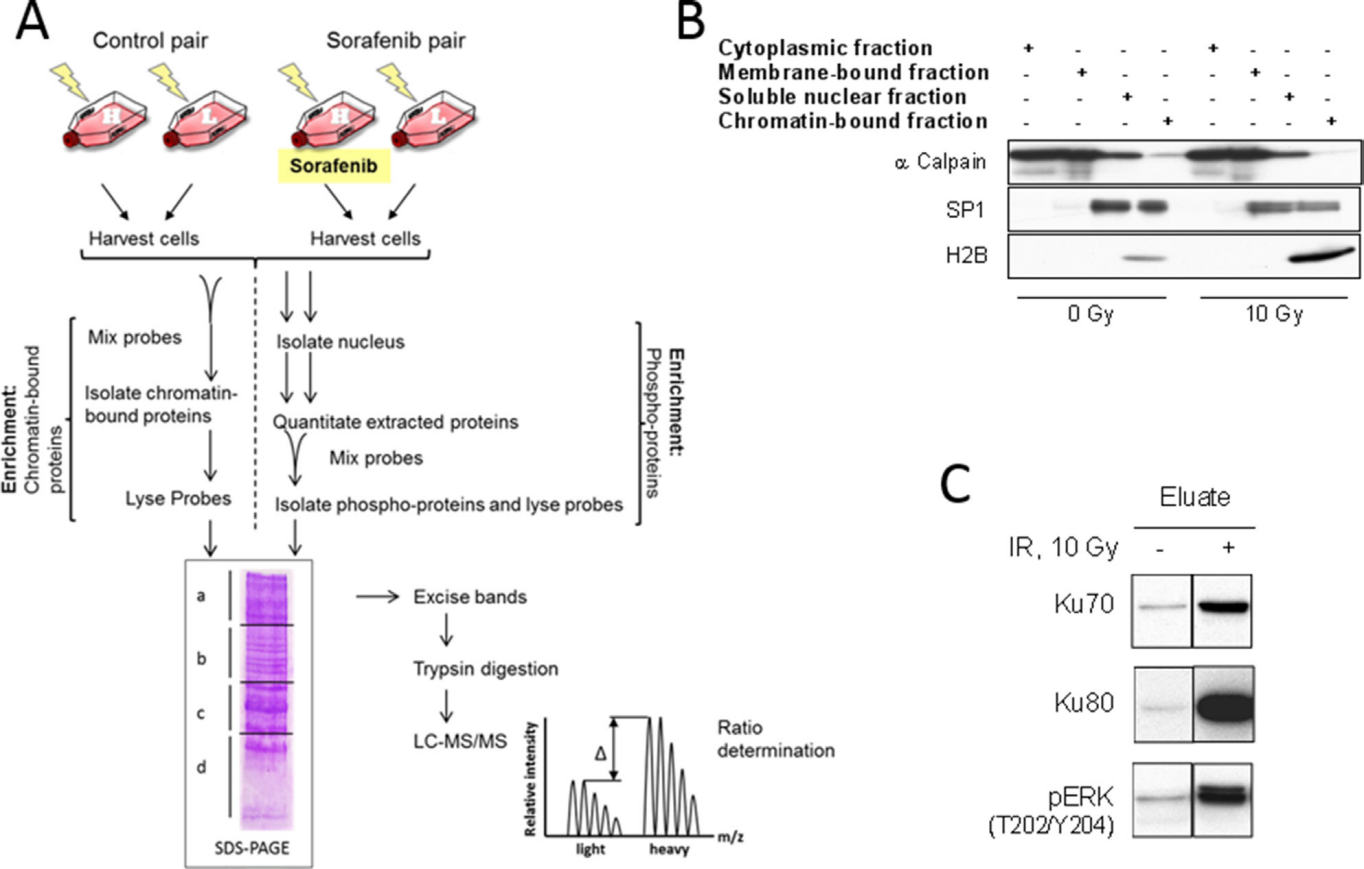
Work flow and sample preparation **(A)** Work flow. Cells were cultivated in SILAC medium containing either heavy (H) or light (L) amino acids. A pair of H- and L- cells were irradiated with 10 Gy (*Control*). Another pair of H- and L-cells was incubated for 2 h with 10 µM sorafenib (H) or DMSO (L) before irradiation (*Probe*). To analyze chromatin-interacting proteins equal numbers of cells were mixed and the chromatin fraction was isolated. To analyze phospho-proteins the nuclei were lysed and equal amounts of proteins were mixed and subjected to affinity purification. The samples were separated via SDS-PAGE and lanes were cut into four fractions, proteins were extracted and digested by trypsin. The peptides from fractions 1–3 (> 98–34 kDa) were quantitatively analyzed using LC-MS/MS. (**B**) Western blot of cell fractionation. αCalpain served as a marker for the non-nuclear fraction, Sp1 as marker for the soluble and the insoluble nuclear fraction and histone H2B as marker for the insoluble chromatin fraction. (**C**) Western blot detecting Ku70, Ku80 and phosphorylated ERK in the phospho-protein eluate.

To analyze sorafenib-induced changes in the chromatin binding or the phosphorylation of nuclear proteins, two protocols were used as depicted in Figure 4A: For both protocols the SILAC-labeled cells were treated with sorafenib (H) or DMSO (L) 2 h before irradiation with 10 Gy and harvested 30 min later. To determine the chromatin-bound fraction, the cells were trypsinized, counted and mixed in equal numbers. This mixture (*Probe*_*Chromatin*_) was lysed and the chromatin fraction isolated (Figure 4B) and analyzed by LC-MS/MS. To examine the fraction of phospho-proteins, the nuclei of sorafenib (H) and DMSO (L) treated cells were isolated, lysed and equal amounts of protein mixed (*Probe*_*Phospho*_). The phospho-proteins were isolated (Figure 4C) and also analyzed by LC-MS/MS. Before the LC-MS/MS all samples were separated into four distinct fractions by SDS-PAGE to reduce complexity: fraction a) > 98 kDa; b) 98–50 kDa; c) 50–34 kDa and d) below 34 kDa. Only the proteins from fractions a-c (> 98–34 kDa) were analyzed. To exclude changes due to SILAC cultivation, control pairs were prepared consisting of heavy and light cells which were irradiated only. These samples are labelled *Control*_*Chromatin*_ and *Control*_*Phospho*_ (Figure 4A).

For the *Probe*_*Chromatin*_ 1096 peptides were identified with 749 peptides being also quantified (*Control*_*Chromatin*_: 524 identified, 307 also quantified). For the *Probe*_*Phospho*_ 552 peptides were identified with 227 peptides being also quantified (*Control*_*Phospho*_: 541 identified, 231 also quantified; See Supplementary Tables 1 and 2). After excluding albumin and keratin the peptides led to the identification of approximately 100 proteins in each sample as depicted in Table 1. On average two third of the identified proteins could also be quantified.

**Table 1:** Number of identified and quantified proteins

	Chromatin-bound proteins		Nuclear phospho-proteins	
Number of proteins	Control	Probe	Control	Probe
Identified	100	133	104	91
Quantified	72	90	62	59
Identified / Quantified^1^	57		47	
(involved in DNA repair)	(4)		(9)	

^1^Identified and quantified in the *Control* and the *Probe*.

**Figure 5:**
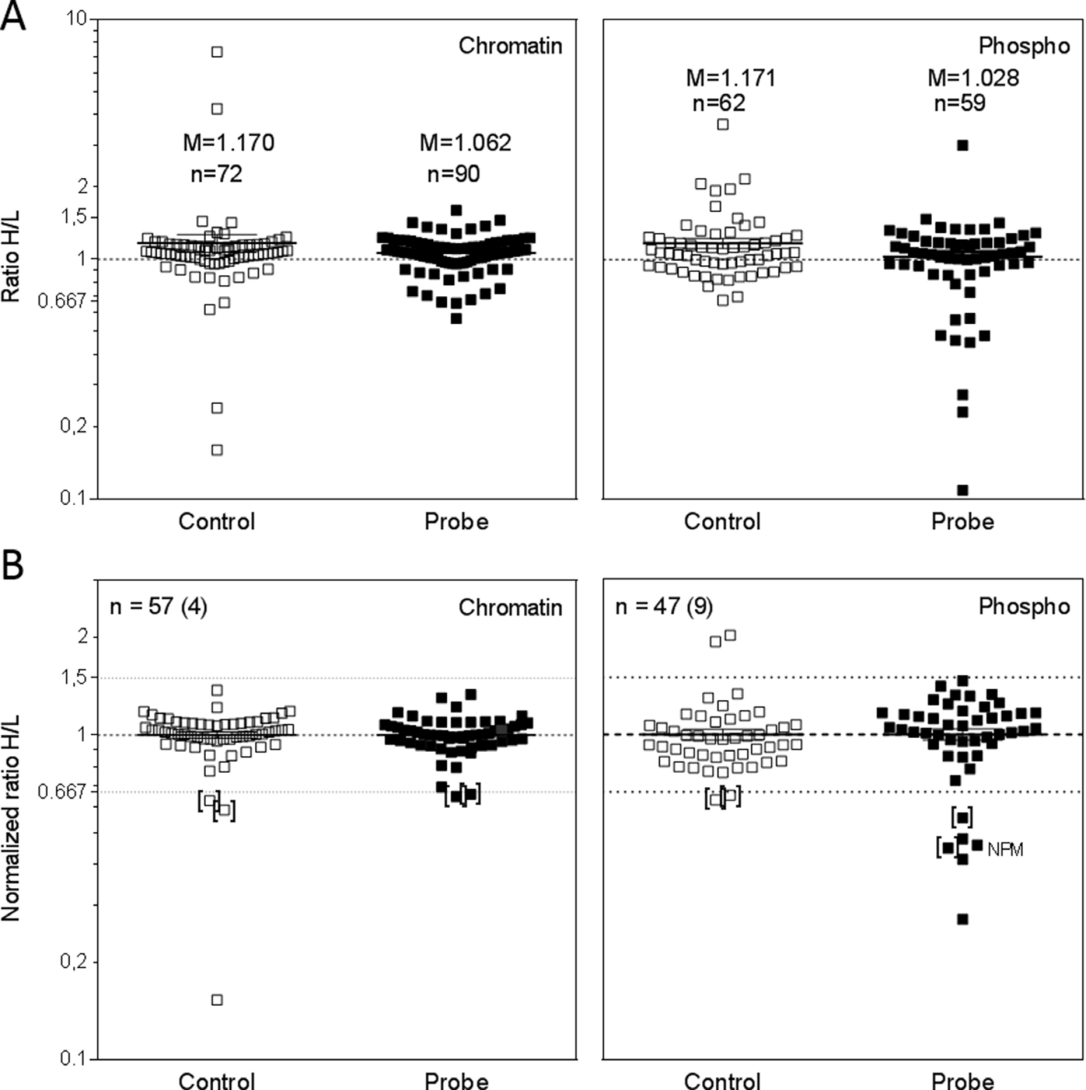
Quantitative analysis of chromatin binding and phosphorylation of nuclear proteins by SILAC-MS (**A**) Identified and quantified proteins in the Control and Probe fractions of chromatin- bound proteins and nuclear phospho-proteins. Depicted is also the number of proteins (*n*) and the mean of the H/L ratios (M). (**B**) Normalized H/L ratios of all proteins detected in both the Control and the Probe of either the chromatin-bound or the nuclear phospho-protein fraction. The number of proteins is indicated (*n*) including the number of DNA repair-related proteins (round brackets). Thresholds for regulation were set at 1.5 (up-regulation) and 0.667 (down-regulation). Proteins which were regulated in the Control and the Probe were bracketed.

Figure 5A shows the dot plots for all quantified proteins in all samples, the *Control* and *Probe* of the chromatin-bound (left) and the phospho-protein fraction (right). In order not to miss any promising proteins, we included also those proteins that were identified and quantified based on none-unique peptides (Supplementary Tables 3 and 4).

In Figure 5B only those proteins were included, which were detected in both the *Probe* and the corresponding *Control*, with *n* indicating the number of proteins; 57 proteins in the chromatin fraction and 47 in the phospho-protein fraction. Focusing on proteins with known functions in DNA damage detection and repair, 4 (chromatin fraction) and 9 (phospho-protein fraction) proteins could be analyzed. Since small variations in the mixing of the samples already affect H/L-ratios we additionally normalized the H/L-values by using the mean H/L-ratios of the individual sample (M: *Control*_*Chromatin*_= 1.050; *Probe*_*Chromatin*_= 1.093; *Control*_*Phospho*_= 1.074; *Probe*_*Phospho*_= 1.009). After setting the thresholds for up – and downregulation at 1.5 and 0.667, respectively, we detected two downregulated proteins in the chromatin fraction and six in the phospho-protein fraction (Table 2). However, for both fractions two of these proteins were also downregulated in the *Controls* (brackets) resulting in no distinct changes in the chromatin fraction but four distinct downregulated proteins in the phospho-protein fraction including the DNA repair protein NPM1.

**Table 2:** Regulated proteins

	Chromatin-bound proteins		Nuclear phospho-proteins	
Protein	Control	Probe	Control	Probe
ACTB^1^	-	-	0,8697845	0,4133276
ACTBL	0,5880827	0,6573057	0,6298223	0,4779822
ACTBM^1^	0,6277214	0,6463761	0,6510096	0,447166
HNRPQ^1^	-	-	1,049211	0,554755
NPM	-	-	1,116355	0,4569528
ROAA	-	-	2,021377	0,2702799

^1^Quantified with none-unique peptides.

Interestingly, the overlap of proteins detected in the chromatin as well as in the phospho-protein fractions was quite high with 40 proteins based on the *Probe* data from Figure 4A and 27 proteins based on the data from Figure 4B.

## DISCUSSION

In this study we introduced and compared two differential proteomic approaches, on one hand the analysis of the chromatin fraction and on the other hand the analysis of the nuclear phospho-protein fraction of cells that had been only irradiated or also treated with sorafenib. Using SILAC-based MS we asked if sorafenib induces changes in the nuclear phospho-proteome or the chromatin fraction and which method might be more suitable for detecting kinase inhibitor-induced changes in a quantitative manner. These changes could serve as pointers for future studies to help unveil the molecular mechanisms underpinning DNA DSB repair inhibition and radiosensitization. The great advantage of this project is the combination of differential proteomics with different enrichment protocols, which allow the examination of clinically relevant problems. Our data support the notion that such functional quantitative MS approaches are indeed very promising tools to unveil the molecular effects of kinase inhibitors on DDR and DNA repair. Comparing both techniques, the overlap of detected and quantified proteins was approximately 50%. However, sorafenib-induced changes were detectable especially in the phospho-protein fraction. This indicates that analyzing the nuclear phospho-proteome is more promising than analyzing chromatin-bound proteins. Besides proteins involved in gene transcription, RNA processing and translation we detected several proteins involved in replication, chromatin remodeling, DDR and DNA repair (see Supplementary Tables 3 and 4). DNA repair proteins included less prominent factors like ILF2, ILF3 and MATR3 but also well-known ones such as PARP1, Ku70 (XRCC5) and Ku80 (XRCC6). Overall, more DNA repair proteins were detected in the phospho-protein fraction. This might be due to the fact, that MS primarily detects the most abundant proteins. Compared to most chemotherapeutics, IR induces only limited numbers of DNA lesions at equitoxic doses, and only one or a few of the individual DNA repair proteins are recruited to the site of damage [16]. Although we used a high dose of IR, 10 Gy might still be insufficient to recruit enough DNA repair proteins to the DNA to be detectable. Nevertheless, with NPM1 we also detected a DNA repair protein, which was definitely regulated in the phospho-protein fraction after sorafenib treatment. Whether DNA repair inhibition by sorafenib is indeed mediated by NPM1 will be investigated in future studies.

Limitations of this study are the relatively small amount of protein which was accessible for the analysis (200 µg was used for the enrichment experiment and approximately 60 µg were loaded on the SDS-PAGE) and the fact, that the Q-TOF Premier which was used generates only 1 precursor spectrum and 2 fragment spectra in 3s. Modern Orbitrap and Q-TOF instruments generate more than 100 fragment spectra within 3s resulting in higher numbers of identified peptides and proteins in recent studies. Furthermore, precipitation intact proteins and not peptides will also result in a relatively low number of identified proteins. But, because peptide precipitation is not suitable for chromatin fraction analysis, we decided to enrich intact proteins. Another factor which reduces the number proteins is the fact that we focused only on proteins which were quantified in both, the *Control* and the *Probe* (compare Figure 5A and 5B). However, the importance of such a *Control* in order to detect false positives or negatives is highlighted by the finding that in all MS measurements the proteins ACTBL and ACTBM were down-regulated in the heavy samples (see Table 2). This indicates a biological response to the heavy amino acids, which can be detected only by including an adequate control. In future studies the number of quantified proteins might be increased by including also proteins smaller than 34 kDa, by an increased LC column length and by further reduction of the complexity of each sample (for example reduced size range of the SDS gel fractions [14] or by isoelectric focusing of the lysates [17]). Another way to increase the number of quantified proteins would be to decrease the cut-off of the SILACAnalyzer. However, this can result in a number of false-positive quantifications and parameters have to be optimized carefully.

By analyzing the nuclear phospho–proteome using SILAC-based MS we have recently identified the molecular mechanism of EGFR-mediated DNA DSB repair inhibition in irradiated HNSCC cells [14]. In that study, erlotinib treatment led to multiple changes in the phospho-proteome but not in the composition of the chromatin fraction (unpublished data [18]). This supports our conclusion that analysis of the nuclear phospho-protein fraction is more promising than that of the chromatin fraction for the detection of interactions of kinase inhibitors with DDR and DNA repair. In this context it has to be mentioned that direct inhibition of DDR-related kinases by kinase inhibitors such as erlotinib or sorafenib cannot be excluded, but an indirect effect is more likely.

In summary, we have demonstrated here, that differential proteomics is a worthwhile tool to investigate the molecular effects of kinase inhibitors on DDR and DNA repair. In this context, the MS-based analysis of the nuclear phospho-proteome might be more promising compared to the analysis of changes within the chromatin fraction.

## MATERIALS AND METHODS

### Cells and cultivation

The HNSCC cell line UT-SCC 42B was grown in D-MEM (Sigma) containing 10% FBS (Biochrom) at 37°C, 10% CO_2_ and 100% humidification. For SILAC experiments cells were cultivated in SILAC-specific D-MEM media containing 10% dialyzed FBS, 200 mg/l proline and 100 mg/l of either [^12^C_6_] L-arginine/[^12^C_6_] L-lysine (light) or [^13^C_6_] L-arginine/[^13^C_6_] L-lysine (heavy, Thermo Scientific). The cell number was quantified using a Coulter-Counter (Beckmann).

### Substances

Small molecule inhibitor: sorafenib (sorafenib tosylate, Nexavar^®^, Bayer HealthCare); DMSO (vehicle, Sigma-Aldrich).

### Irradiation

Cells were irradiated at room temperature with 200 kV X-rays (Gulmay RS225, Gulmay Medical Ltd.: 15 mA, 0.8 mm Be + 0.5 mm Cu filtering; dose rate of 1.2 Gy/min).

### Immunofluorescence and analysis of DNA repair

Residual γH2AX/53BP1 double positive repair complexes (foci) were analyzed as described earlier [8]. In brief, 1.5 x 10^5^ cells were seeded onto culture slides (BD-Falcon), treated 24 h later and irradiated with 2 Gy 2 h thereafter. Another 24 h later cells replated and were fixed, permeabilized, blocked and incubated with anti-γH2AX (Merck Millipore, 05–636) and anti-53BP1 (Novus Biologicals) primary and secondary antibodies (Fluorescein-labeled anti-mouse: red, life technologies, A11005; anti-rabbit: green, GE-Healthcare, Amersham™) 24 h later. Cell nuclei were stained with DAPI (QBiogene). The γH2AX/ 53BP1 foci per nucleus were analyzed visually by fluorescence microscopy (Zeiss Axioplan 2) with 630x magnification. At least 100 intact nuclei were randomly chosen for the evaluation. Experiments were repeated four times.

### Isolation of chromatin-bound proteins

The *Subcellular Protein Fractionation Kit* (Thermo Scientific) was used according to the producer´s instruction. In brief, trypsinized cells were centrifuged (5 min, 500 g, 4°C) and 2 × 10^6^ cells were incubated with 100 µl CEB buffer and gently mixed for 10 min on a rotator at 4°C. All buffers contained phosphatase/protease inhibitors (Halt inhibitor cocktail, Thermo Scientific). After centrifugation (5 min, 500 g, 4°C) the supernatant was collected (cytoplasmic proteins). The cell pellet was consecutively incubated with 100 µl MEB (membrane-bound proteins), 50 µl NEB (soluble nuclear proteins) and 50 µl NEB with 5 µl CaCl_2_ (100 mM) and 3 µl MNnase (300 U) (chromatin-bound proteins) with respective centrifugation.

### Isolation of phospho-proteins

Plateau phase cells were harvested mechanically, resuspended in PBS and centrifuged (5 min, 1000 g, 4°C). The cell pellet was resuspended in 500 µl hypotonic lysis-buffer (10 mM HEPES pH 7.9, 10 mM KCL, 0.1 mM EDTA, 0.1 mM EGTA, phosphatase/protease inhibitors (Halt inhibitor cocktail, Thermo Scientific)) and incubated for 15 min on ice. NP-40 was added to a final concentration of 0.625%, the sample mixed for 10 s and centrifuged for 10 min (1000 g, 4°C). The pellet containing the nuclei (supernatant, cytoplasmic fraction) was resuspended in 500 µl Extraction/ Loading Buffer (Clontech), incubated on ice for 20 min and mixed periodically. After centrifugation (20 min, 20,000 g, 4°C) the supernatant (nuclear fraction) was treated with ultrasound for 10 s and used for phospho-protein isolation, which was performed using the *TALON*^®^*PMAC Magnetic Phospho Enrichment Kit* (Clontech) according to the producer´s instruction. In brief, 200 µg nuclear protein lysates were incubated with 200 µl magnetic beads and 10 mM NaF for 1 h at 4°C while gently mixing on a rotator. The supernatant was collected. After washing the beads three times the phosphorylated proteins were eluted with 50 µl elution buffer.

### Protein quantification

Nuclear protein concentration was quantified using bicinchoninic acid assay (BCA, Sigma) according to the producer´s instruction.

### Western blot

Proteins were detected by Western blot according to the standard protocol. Primary antibodies from Cell Signaling Technology: anti-Ku70 (rabbit, #4104), anti-Ku80 (rabbit, #2753), anti-pERK1/2 (Thr202/Tyr204, rabbit, #4370); from Merck Millipore: anti-calpain (mouse, 208730); from Santa Cruz Biotechnology: anti-SP1 (rabbit, sc14027) and from Imgenex: anti-H2B (rabbit, #IMG-359). Secondary antibodies from Amersham: anti-rabbit and anti-mouse HRP-conjugated. Signals were determined by chemiluminescence using the ECL™ Western Blotting Detection Reagents (Amersham).

### LC-MS/MS analysis

Proteins were separated via SDS-PAGE using a 15% Bis-Tris. The gels were stained with Coomassie blue for 30 min followed by destaining. Each lane was separated into four fractions (a => 98 kDa; b = 98–50 kDa; c = 50–34 kDa and d = < 34 kDa). For fraction a-c a tryptic in-gel digestion was performed in accordance to Shevchenko et al. [19]. After digestion, the samples were evaporated and mass spectrometric proteome analysis was performed by injecting the samples on a nano-ultra pressure liquid chromatography system (nano-UPLC; nanoACQUITY, Waters, Manchester, UK) coupled via electrospray-ionization (ESI) to a quadrupole time-of-flight (QTOF) mass spectrometer (QTOF Premier, Micromass/Waters, Manchester, UK) as described by Kwiatkowski et al. [20] with slight modifications. After sample loading on a trapping column (nanoAcquity UPLC PST trap column, C18, 180 μm × 20 mm, 5 µm, 100 Ǻ, Waters, Manchester, UK; buffer A: 0.1% FA in HPLC-H2O; buffer B: 0.1% FA in ACN), peptides were eluted onto the separation column (nanoAcquity UPLC BEH column, C18, 75 μm × 150 mm, 100 Ǻ Waters, Manchester, UK; 200 nL/min, gradient: 2−35% B in 30 min). The spray was generated from a fused-silica emitter (I.D. 10 μm, New Objective, Woburn, USA) at a capillary voltage of 1650 V, a source temperature of 100°C and a cone voltage of 40 V in positive ion mode. For MS/MS measurements, data were recorded in the data dependent acquisition mode (DDA). MS survey scans were performed over an m/z range from 400–1300 with a scan-time of 0.6 s and an interscan delay of 0.05 s. The two most abundant signals were used for fragmentation. MS/MS spectra were obtained from 100–1500 m/z with a scan-time of 0.95 s and a collision ramp from 22–30 eV. An online exclusion was used to prevent multiple fragmentation events (exclusion time: 20 s, exclusion window: +/− 2 m/z).

### Data analysis of LC-MS/MS data

Data analysis was performed as described earlier [14, 17] with slight modifications. Briefly, data from LC-MS/MS analysis were analyzed using the open-source software framework OpenMS [21] and the OpenMS Proteomic Pipeline (TOPPAS) [22]. For peptide and protein identification, mzML-files were searched against a human decoy-database (Swiss-Prot, www.uniprot.org, 20,161 entries) using two different search engines (open mass spectrometry search algorithm (OMSSA) [23] XTANDEM [24]). The search was performed with a precursor mass tolerance of 35 ppm and a fragment mass tolerance of 0.1 Da. Carbamidomethylation on cystein residues was considered as a fixed modification. An oxidation of methionine residues as well as a ^13^C_6_-label on arginine and lysine residues were considered as variable modifications. Peptides were identified with a q-value of 0.05. For SILAC quantification the raw data files were converted to ^*^.mzXML in profile mode using massWolf file converter. Further data processing was carried out with TOPPAS. The ^*^.mzXML files were converted to ^*^.mzML. For subsequent data processing the mzML files were filtered (only MS1 level, rt-range [s]: 600–2400) and smoothed (savitsky golay algorithm, frame length: 13, polynomial order: 4). SILAC pairs were detected and quantified using SILACAnalyzer with the following parameters: one missed cleavage, retention time threshold 10 s, intensity cutoff 20 counts, intensity correlation 0.7 and a model deviation of 1.8. A peptide required at least three isotopic peaks and maximal seven isotopic peaks to be taken into account by the SILACAnalyzer. Detected SILAC pairs were exported as ^*^.consensusXML and matched with peptide identifications (^*^idXML) using IDMapper (retention time tolerance: 10 s, m/z-tolerance: 1 Da). Results were exported as ^*^.csv and further statistical processing was carried out using mathematica.

### Data evaluation

Microsoft Excel and Prism 5 software (GraphPad Software) software were used for analyzing and graphing the data. Except for the mass spec experiments, the data were repeated at least three times and are presented as mean values (+/-SEM). The Prism 5 software was used for analyzing and graphing the data. The paired student’s *t*-test was performed for the statistical analysis. *P*-values were calculated using two-sided tests (^*^p < 0.05; ^**^p < 0.01; ^***^p < 0.001).

## SUPPLEMENTARY MATERIALS TABLES










