# Supplementary material for: Analyzing the influence of kinase inhibitors on DNA repair by differential proteomics of chromatin-interacting proteins and nuclear phospho-proteins
Source: Oncotarget. 2017 Nov 10;8(67):110983–93. doi: 10.18632/oncotarget.22424 (PMC5762299; doi:10.18632/oncotarget.22424)
Supplement: Supplementary file 1 [file oncotarget-08-110983-s001.pdf]

Analyzing the influence of kinase inhibitors on DNA repair by differential proteomics of chromatin-interacting proteins and nuclear phospho-proteins

SUPPLEMENTARY MATERIALS

Supplementary Table 1: Peptide list for chromatin fraction control and probe. See Supplementary_Table_1

Supplementary Table 2: Peptide list for phospho protein fraction control and probe. See Supplementary_Table_2

Supplementary Table 3: Protein list for chromatin fraction Control and Probe. See Supplementary_Table_3

Supplementary Table 4: Protein list for phospho protein fraction Control and Probe. See Supplementary_Table_4
